# Advancements and Applications of Lateral Flow Assays (LFAs): A Comprehensive Review

**DOI:** 10.3390/s25175414

**Published:** 2025-09-02

**Authors:** Dickson Mwenda Kinyua, Daniel Maitethia Memeu, Cynthia Nyambura Mugo Mwenda, Bartolomeo Della Ventura, Raffaele Velotta

**Affiliations:** 1 Department of Pure and Applied Sciences, Kirinyaga University, Kerugoya 143-10300, Kenya; 2 Department of Physical Sciences, Meru University of Science and Technology, Meru 972-60200, Kenya; 3 Department of Biological Sciences, Meru University of Science and Technology, Meru 972-60200, Kenya; 4 Department of Physics “Ettore Pancini”, University of Naples Federico II, 80126 Naples, Italy

**Keywords:** lateral flow assays (LFAs), point-of-care diagnostics, artificial intelligence, nanoparticles, multiplexing, limit of detection, sensitivity, colorimetric

## Abstract

Over a decade ago, WHO introduced the ASSURED (Affordable, Sensitive, Specific, User-friendly, Rapid and Robust, Equipment-free, and Deliverable to end-users) criteria to guide diagnostic assay development. Today, lateral flow assays (LFAs) best meet these standards, evolving from simple rapid tests to advanced diagnostics integrating AI and nanotechnology for precise, quantitative results. Notably, nanoparticle-enhanced LFAs have achieved limits of detection (LOD) as low as 0.01 pg/mL (a 100-fold improvement over conventional methods), while AI algorithms have reduced interpretation errors by 40% in low-contrast conditions. The COVID-19 pandemic underscored the societal impact of LFAs, with over 3 billion antigen tests deployed globally, demonstrating 98% specificity in real-world surveillance. Beyond infectious diseases, LFAs are revolutionizing cancer screening through liquid biopsy, achieving a 92% concordance rate with gold-standard assays, food safety and environmental monitoring. Despite these advancements, challenges remain in scalability, reproducibility, sustainable manufacturing, and how to enhance the sensitivities and lower the LOD. However, innovations in biodegradable materials, roll-to-roll printing, CRISPR-integrated multiplexing, and efficient functionalization methods like photochemical immobilization technique offer promising solutions, with projected further cost reductions and scalability. This review highlights the technological evolution, diverse applications, and future trajectories of LFAs, highlighting their critical role in democratizing diagnostics.

## 1. Introduction

**Figure 1:**
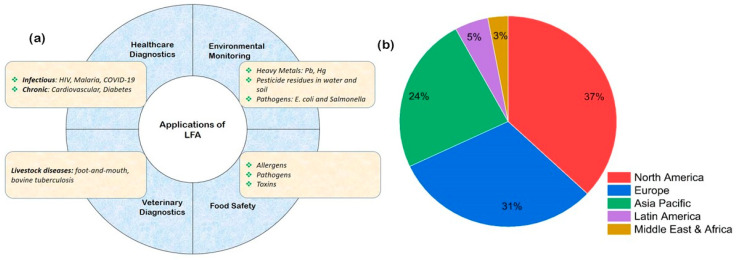
(**a**) Schematic representation of the applications of LFAs in several key areas, demonstrating their versatility and ability to revolutionize the landscape of point-of-care testing and rapid diagnostics. (**b**) LFA market share by region, which shows low uptake in LMICs despite the higher levels of burden of disease in these countries [4].

In the realm of diagnostic technologies, lateral flow assays (LFAs) have emerged as versatile and powerful tools, revolutionizing the landscape of point-of-care testing and rapid diagnostics. Over the years, substantial advancements in both design and functionality have propelled LFAs beyond their initial use cases, expanding their applications across diverse fields such as healthcare, environmental monitoring, food safety, and biotechnology. Figure 1a details the applications of LFAs in several key areas. This comprehensive review aims to explore the recent innovations in LFA technology and delve into the wide-ranging applications that have solidified its status as a cornerstone in the field of diagnostics. To understand the current state of LFAs, it is crucial to trace their historical roots, which is a culmination of several converging advancements that trace back to the 1950s [1,2]. However, significant advancements in this area occurred in the 1970s, driven by improvements in antibody generation technologies and a deeper understanding of the biology and detection of human chorionic gonadotropin (hCG), with the main application driving the early development of the rapid-test technology being the human pregnancy test [3].

The full development of LFA technologies required the integration of various enabling technologies. These inventions include nitrocellulose membrane manufacturing, antibody generation, fluid dispensing and processing equipment, and the accumulation of knowledge in development and manufacturing methodologies. These elements were essential to transform a complex mixture of chemicals, biologicals, papers, polymers, people, and processes into a simple and easy-to-use test capable of providing accurate prognostic results across a range of critical applications [5]. Consequently, the first LFA products entered the market in the late 1980s. Since then, the technology, its applications, and the industry have continuously evolved. As of 2024, the global lateral flow assay market size accounted for USD 16.8 billion and is expected to reach around USD 24.39 billion by 2034 [4]. However, the uptake of these devices in low- and middle-income countries (LMICs) is still low (see Figure 1b). This is despite the fact that, around 70% of healthcare decisions rely on diagnostic test results, and only 3–5% of healthcare budgets are allocated to diagnostic services in these countries [6]. In LMICs, clinicians lack access to essential diagnostic tools and laboratory tests necessary for patient care. Even when these resources are available, geographical barriers and remote communities often make testing a significant challenge. Lateral flow assays (LFAs) present a viable and cost-effective solution to address these challenges. This implies more needs to be performed to make these devices affordable and accessible because they provide the best bet to empower local healthcare providers by enabling decentralized testing and reducing the need for patients to travel long distances for diagnosis.

LFAs typically consist of a porous membrane, commonly made from nitrocellulose, along with a sample pad, conjugate pad, detection zone, and absorbent pad. The combination of these components, along with the choice of materials, determines the assay’s performance characteristics. Recent advancements in material science and engineering have led to the development of novel membranes, conjugates, and detection strategies, resulting in improved sensitivity and specificity [7]. One of the notable breakthroughs in LFA technology is the ability to perform multiplexed analyses, enabling the simultaneous detection of multiple analytes within a single test [8,9,10]. This capability has significantly expanded the scope of LFAs, allowing for comprehensive diagnostic assessments and profiling [11]. Researchers have explored various strategies, including the use of different detection labels, to achieve multiplexing without compromising assay performance. Furthermore, signal enhancement techniques play a pivotal role in pushing the limits of LFA sensitivity. Innovations such as the incorporation of nanoparticles [12,13,14], signal amplification strategies [15,16,17,18], and the integration of smartphone-based detection systems have markedly improved the limit of detection (LOD) for various analytes [16,17,18]. These developments are particularly crucial for applications where early and accurate detection is paramount, such as infectious diseases and cancer diagnostics.

LFAs have transcended their origins in pregnancy testing and are now employed across a myriad of applications. In healthcare, LFAs are instrumental in the rapid diagnosis of infectious diseases, cardiac markers, and chronic conditions. Environmental monitoring benefits from LFAs for on-site detection of contaminants, while the food industry relies on LFAs for rapid and cost-effective screening of pathogens and allergens. As LFAs continue to evolve, future directions include the integration of artificial intelligence for result interpretation, the development of more sustainable and environmentally friendly materials, and further advancements in multiplexed detection. Despite these exciting prospects, challenges such as standardization, assay reproducibility, and overcoming matrix effects persist and warrant ongoing research efforts. From simple use of pregnancy testing to multifaceted applications in healthcare, environmental monitoring, and beyond, LFAs have become indispensable tools for rapid and reliable point-of-care testing. This comprehensive review explores recent innovations in lateral flow assay (LFA) technology and examines its diverse applications, which have cemented its role as a fundamental tool in diagnostics. Additionally, it provides insights into emerging trends and future advancements, highlighting the potential of LFAs to further revolutionize point-of-care testing and expand their impact in global healthcare.

## 2. Principles of Lateral Flow Assays

**Figure 2:**
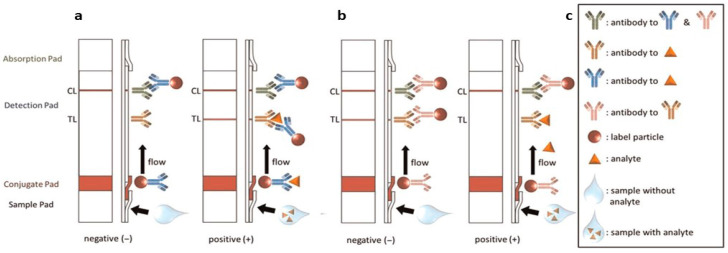
(**a**) Schematic illustration of the binding interactions in a conventional lateral flow assay (LFA), showing the events occurring at the conjugate pad, test line, and control line during target analyte detection. (**b**) Diagram depicting the structural layout of a competitive LFA format, highlighting differences in assay design. (**c**) Legend identifying key components and molecular interactions shown in panels (**a**,**b**). (Reproduced with permission from [9] copyright 2025, Elsevier).

LFAs operate on the principles of immunochromatography, relying on the specific binding of target analytes to antibodies or other biomolecules immobilized on a porous membrane. The key components of an LFA include a sample pad, conjugate pad, nitrocellulose membrane, absorbent pad, and a backing material. The flow of fluids through these components is driven by capillary action. Key steps in the LFA process include sample application, conjugate release, target binding, and detection, as shown in Figure 2a) and detailed as follows [19]: A liquid sample is introduced to the sample pad located directly beneath the sample well. The sample wicks along the LFA membrane to the conjugate pad, where the analyte (if present in the sample) will be linked or binds to the label particles previously functionalized with a bioreceptor specific to the analyte. The bioreceptor is typically an antibody (Abs) that either binds to the target antigen or is functionalized to a detection medium, such as colloidal gold. To prevent interference with test line binding, functionalized Abs are designed to bind to different regions of the target antigen than the test line Abs. When detecting Abs, the functionalized Abs typically binds to the constant region of the target antibody rather than the variable region specific to the target.

When the sample reaches the control line (CL), a binding reaction occurs to confirm the successful release of the conjugate and the transfer of the sample along the membrane. This binding typically is independent of the sample analyte, meaning a CL will appear even if the target analyte is absent. Finally, the remaining sample flows into the absorption pad at the end of the LFA. After applying the sample, the test must sit for a specified amount of time to allow the binding and detection reactions to occur. However, there is also a variant LFA typically known as the competitive model where the analyte and label particles compete to be captured on the test line (TL), obtaining a response inversely proportional to the concentration of analyte (see Figure 2b). Optical detection, particularly colorimetric detection, is most commonly used, as the results can be read by the eye. However, some assays use fluorescence detection, which requires a specialized reader [17,18,20,21,22]. Surface-enhanced Raman spectroscopy (SERS) [22], electrochemical [22,23,24] and magnetic label [25,26,27] detection methods. The choice of conjugate label determines the required detection modality.

## 3. Recent Advancements in LFA Technology

Recent developments in lateral flow assay (LFA) technology have significantly improved sensitivity, specificity, and multiplexing capabilities. These advancements have led to more accurate and reliable tests that can detect lower concentrations of analytes, distinguish between closely related substances, and simultaneously identify multiple targets in a single assay. Innovations in materials, such as enhanced membrane and conjugate designs, as well as improved detection techniques, have contributed to these enhancements. Additionally, integration with digital and mobile technologies has facilitated real-time data analysis and remote diagnostics, further expanding the applications and accessibility of LFA technology in various fields, including healthcare, environmental monitoring, and food safety. These innovations include the following:

### 3.1. Sensitivity Enhancement Strategies in Lateral Flow Assays (LFAs)

Signal amplification aims to increase the detectable signal generated per binding event. This includes nanoparticle-based enhancement, enzyme-mediated amplification, and nanozymes. The incorporation of nanoparticles such as gold and carbon nanoparticles and quantum dots as labeling agents in lateral LFAs has significantly enhanced signal amplification and improved detection sensitivity. Although a variety of signal readout strategies have been developed, optical detection remains the most widely utilized approach due to its simplicity and versatility. This includes both qualitative or semi-quantitative visual interpretation by the naked eye and more sensitive, instrument-assisted measurements. Colorimetric detection in LFAs leverages changes in the local refractive index and plasmonic coupling between nanoparticles to generate visible color shifts [28,29]. Among the various nanomaterials employed, gold nanoparticles (AuNPs) are the most widely used colorimetric labels in LFAs. They have well-established synthesis protocols, are time-stable, size-tunable, biocompatible and have excellent optical properties particularly their intense localized surface plasmon resonance (LSPR) [10,30]. Furthermore, AuNPs offer a variety of surface functionalization protocols [31] and widespread use as a standard labeling material in both commercial and research-based LFA platforms. This section primarily discusses enhancement strategies aimed at improving the analytical performance of LFAs, with a particular focus on systems utilizing gold nanoparticles (AuNPs), given their numerous advantages outlined above. Fundamentally, the intensity of the red coloration at the test line is directly correlated with the concentration of the target analyte present in the sample, serving as a semi-quantitative or quantitative indicator depending on the detection method employed.

A key strategy used to enhance the sensitivity of the LFA is to surround AuNPs with other compounds that enhance the color, enabling more spectral resolution. Mao et al. used horseradish peroxidase (HRP)-AuNP dual labels for the detection of human genomic DNA directly with a detection limit of 2.5 µg/mL (1.25 fM) by adopting well-designed DNA probes [32]. In the development of multianalyte LFA for semi-quantification by the naked eye, the use of HRP-AuNPs dual labels enhanced the detection limits to 10 μg/L for carbaryl and 1 μg/L for endosulfan compared to 100 and 10 μg/L in gold-based assays, respectively [33]. Parolo et al. [28] tested two detection strategies: one following just the red color of the AuNPs and the second one using a substrate for the HRP to enhance the intensity of the previous red color of the unmodified AuNPs. The use of HRP enhanced the sensitivity by an order of magnitude. A combination of monoclonal antibody and the platinum gold nanoflower core-shell (Pt@AuNF) nanozyme and HRP were used to detect Zearalenone. They showed that the limit LOD of the Pt@AuNF-HRP labeled LFA strips after signal amplification was 0.052 ng/mL, and the detection range was 0.052–7.21 ng/mL. This not only expanded the detection range by 5-fold due to improved sensitivity but also reduced the amount of the probe by half to achieve antibody conservation [34].

In general, horseradish peroxidase (HRP) has been demonstrated to significantly improve the sensitivity of various bioassays, including lateral flow assays, by facilitating signal amplification [15,31,32,33,34]. However, the practical application of natural enzymes like HRP is hindered by several intrinsic limitations. These include poor thermal and chemical stability, susceptibility to denaturation over extended storage periods, or upon exposure to harsh environmental factors. As a result, there is growing interest in the development and optimization of artificial enzymes that can replicate or even surpass the functionality of natural enzymes while offering superior robustness, shelf stability, and cost-effectiveness. These efforts are geared towards the development of high-performance bioanalytical platforms and are particularly promising for improving the reliability and durability of point-of-care diagnostics.

**Figure 3:**
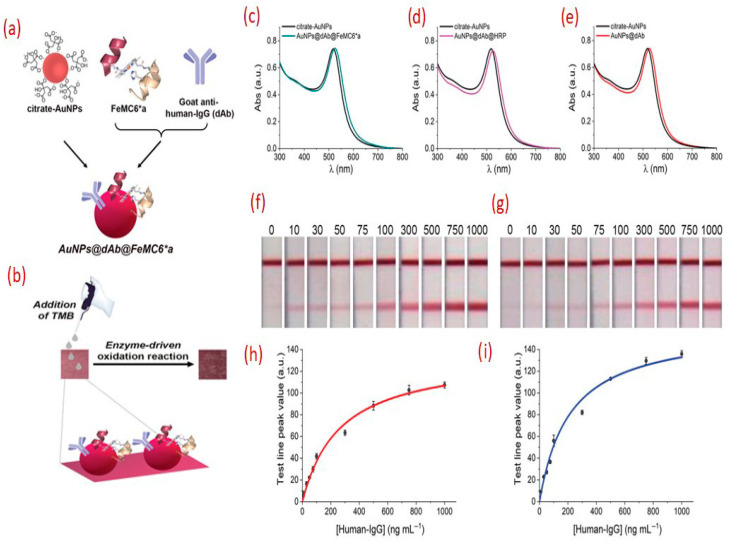
(**a**) Schematic illustration of the dual-labeled AuNPs@dAb@FeMC6a conjugates employed in the lateral flow assay (LFA). (**b**) Conceptual diagram showing the enhancement of test line (TL) signal intensity through catalytic oxidation of the TMB chromogen by FeMC6a, resulting in a darker color and improved assay sensitivity. UV–Vis spectra of citrate-stabilized AuNPs (black curves) and their functionalized forms: (**c**) AuNPs@dAb@FeMC6a, (**d**) AuNPs@dAb@HRP, and (**e**) AuNPs@dAb. The redshift observed in all functionalized samples confirms successful conjugation, with the shift in (**e**) attributed solely to dAb binding. (**f**,**g**) Photographic images of LFA strips using AuNPs@dAb@FeMC6a in a two-step assay format, where TMB is added post-migration to amplify the visual signal. (**h**,**i**) Calibration curves for human IgG detection before (**h**) and after (**i**) the addition of TMB, demonstrating enhanced sensitivity following enzymatic signal amplification(Reproduced with permission from [35], copyright 2025, John Wiley & Sons).

Recently, Renzi et al. demonstrated that the use of the artificial peroxidase FeMC6a conjugated to gold nanoparticles (AuNPs) enabled significantly lower detection compared to those utilizing natural horseradish peroxidase (HRP) [35]. Notably, even further improvements in sensitivity were achieved using a two-step assay format that strategically exploits the catalytic activity of FeMC6a. In this approach, HRP and FeMC6a both catalyze the oxidation of the colorless substrate 3,3′,5,5′-tetramethylbenzidine (TMB), but FeMC6a demonstrated superior performance, producing more than a four-fold enhancement in signal intensity. In contrast, HRP in combination with AuNPs yielded only a two-fold improvement under similar conditions (see Figure 3). The assay operates in two stages: in the first step, red bands appear at the test and control lines due to the accumulation of AuNPs, as observed in conventional AuNP-based LFAs. In the second step, the TMB substrate is introduced and is subsequently oxidized by the peroxidase immobilized on the AuNPs. This enzymatic reaction results in the generation of an additional colored product at the test line, significantly enhancing the overall signal intensity and thereby improving assay sensitivity.

Other ways to enhance sensitivity include replacing visual inspection with sensitive detectors, which can greatly improve LFA performance. Fluorescent LFAs, employing quantum dots, up-conversion nanoparticles, or dye-doped microspheres, achieve markedly lower detection limits and broader dynamic ranges [36]. Similarly, surface-enhanced Raman scattering (SERS) LFAs use Raman-active nanoprobes and portable Raman readers to amplify signal intensity by tens- to hundreds-fold compared to conventional optical LFAs [10]. In general, besides the exceptional sensitivity these methods offer other advantages like quantitative capability and compatibility with multiplexing. However, it should be noted that dependence on external hardware may lead to increased cost and complexity. In addition, pre-assay enrichment can overcome inherent binding kinetics limits. Techniques include sample pre-concentration using filtration or microfluidic concentration and nucleic acid amplification (e.g., recombinase polymerase amplification, loop-mediated isothermal amplification) for pathogen detection [36]. Usually, this results in sensitivity gains, especially for low-abundance targets. Finally, physical modifications to the LFA strip can improve sensitivity by controlling flow and increasing interaction time between analyte and capture probes. For example, altering nitrocellulose pore size, integrating flow-delay barriers, or employing multi-line test formats. Three-dimensional paper-based LFAs increase surface area and allow sequential reagent delivery, boosting capture efficiency [10]. Each of these sensitivity-enhancement strategies presents a balance between analytical performance, cost, ease-of-use, and manufacturability. Physical and chemical amplification methods (e.g., AuNP–HRP, silver enhancement) offer substantial gains with modest complexity, while advanced reader modalities and target amplification provide the highest sensitivity at the expense of simplicity.

### 3.2. Smartphone and Digital Reader Integration in LFA Technology

The integration of smartphones with lateral flow assays (LFAs) has revolutionized diagnostic accessibility, transforming handheld devices into portable laboratories capable of quantitative, real-time analysis. This synergy leverages smartphone cameras as optical sensors and computational power, addressing longstanding limitations in visual interpretation of LFAs [37]. Using apps, signal processing takes various steps, starting from image processing to more advanced machine learning algorithms to quantify line intensity or color change, correlating pixel values to analyte concentration. As an example, an LFA for environmental monitoring was developed to measure Hg(II) and integrated a smartphone camera and a written smartphone application to quantify, achieving a limit of detection (LOD) of 2.53 nM [38]. Smartphone-aided LFA assays have been used as pasteurization indicators to detect alkaline phosphatase in milk with a limit of 0.1 U/L and a dynamic range up to 150 U/L when analyzed by a phone camera [39] and to detect pesticides and antibiotics in produce and dairy, vitamins in blood, and hormones in animal health [40]. With the COVID-19 pandemic, the adoption of smartphone LFA readers has been rapidly propelled, with various research groups and companies availing built apps to guide users on testing and automatically interpret the results [41]. The use of smartphones to aid LFA signal interpolation coupled with deep learning-assisted smartphone apps is especially a critical and transformative aspect of revolutionizing diagnostics, with recent studies by Lee et al. showing that the app achieved ~98% accuracy on 135 clinical COVID LFAs and maintained >99% accuracy for low viral load samples, whereas human readers often missed these weak positives [42]. Other studies in malaria, HIV, and other infectious diseases have shown the potential of smartphone integration to broaden quality healthcare by enabling tests to be instantly shared and geotagged for public health surveillance [41,43]. In addition to smartphone systems, various dedicated hardware readers have been developed [37,44]. For instance, clip-on attachments with 20-megapixel lenses have been shown to improve detection accuracy by 95% compared to unaided visual analysis in malaria diagnostics [45]. On the software front, AI-driven algorithms now dominate signal interpretation, minimizing human error and enabling semi-quantitative results. Convolutional neural networks (CNNs) trained on thousands of LFA images can distinguish faint test lines with 98% specificity, even in low-contrast conditions [46].

In principle, smartphone-based and digital LFA readers are supposed to improve the analytical performance of LFAs by improving the quality of the information available to enable decision making. Therefore, studies on how to enhance key parameters like sensitivity, LOD, accuracy, and inter-operator consistency will pave the way for more robust diagnostic tools, especially in resource-limited settings. Studies have reported that digital analysis can improve LFA sensitivity by several-fold, with some being as simple as optimizing imaging parameters, e.g., using longer camera exposure or algorithmic contrast enhancement to boost signal-to-noise ratio and reveal faint positives that would be missed by the eye. Other than the colorimetric approach, other strategies can be to use more sensitive labels like fluorescent NPs, up-conversion phosphors, and imaging in the infrared or even enzymatic chemiluminescence38. These advancements are not without challenges: device standardization remains elusive, with a 2023 meta-analysis revealing a 30% variability in results across smartphone models under identical lighting [47]. Furthermore, ambient light interference can alter colorimetric readings by up to 40%, necessitating adaptive algorithms or controlled lighting chambers [48], with offered solutions being the use of calibration cards, fixed phone holders, or built-in corrections to standardize the reading conditions [41,43]. Despite these hurdles, the trajectory points toward decentralized diagnostics. Cloud-based platforms like “MediCloud” now aggregate LFA data from rural clinics, enabling real-time outbreak tracking—a strategy deployed during the 2023 dengue surge in Southeast Asia, where 500,000 tests were analyzed remotely within two weeks [49]. Telemedicine integration further expands utility; the “Scan4Health” project in Sub-Saharan Africa pairs smartphone LFAs with clinician consultations, achieving a 50% reduction in tuberculosis diagnostic delays [50]. As 5G networks expand, AI models are shifting to edge computing, allowing offline analysis in resource-limited settings—a critical advance given that 3.6 billion people still lack internet access [51]. With the global smartphone-LFA market projected to reach USD 2.8 billion by 2026 [52] this fusion of consumer technology and diagnostics is poised to democratize precision medicine globally.

### 3.3. Multiplexed LFAs

Multiplexed LFAs represent a transformative leap in diagnostic efficiency, enabling simultaneous detection of multiple analytes within a single test strip. This advancement consolidates several individual tests into one, meeting key requirements of ease of use, low detection limits, and high specificity [53]. Furthermore, multiplexing reduces the sample volume and number of separate assays needed [54]. Spatial multiplexing, which employs distinct test lines for each target, remains the most widely adopted strategy due to its simplicity and compatibility with existing LFA architectures [55,56,57]. For example Norz et al. [58] developed a multiplexed LFA for SARS-CoV-2 and influenza A/B, achieving 97% clinical agreement with PCR across 1200 patient samples. Each test line utilized antibodies specific to nucleocapsid proteins, with a limit of detection (LOD) of 50/mL for SARS-CoV-2, comparable to standalone tests. In food safety, multi-line LFAs have been created to concurrently detect mycotoxins, antibiotic residues, and pesticides in one run [59,60,61,62]. However, spatial designs face inherent limitations in scalability, as increasing test lines beyond 3–4 compromises strip readability and requires wider membranes, raising production costs by up to 40% [63]. Still, studies have demonstrated that it is possible to simultaneously detect 32 different drug molecules in urine by using smaller dot-shaped capture spots in a microarray format, greatly increasing the density of test zones on one strip [64]. The tradeoff was limiting the robustness of the assay result, because the smaller the detection site, the higher the impact of flow inhomogeneity on the generation of the signal. Spectral multiplexing, in contrast, leverages advanced nanomaterials like quantum dots (QDs) or fluorescent dyes to encode multiple targets within a single test line. QD barcoding, for instance, enables simultaneous detection of HIV, syphilis, and hepatitis B markers with distinct emission wavelengths, achieving 92% accuracy in a 2022 meta-analysis of 15 studies [49,65,66]. Sun et al. [67] further refined this approach by integrating orthogonal antibodies—antibodies targeting non-overlapping epitopes—to minimize cross-reactivity, reducing false positives by 80% in multiplexed sepsis biomarker panels. The use of different plasmonic colorimetric NPs can also be employed. For example, a recent allergen test employed three types of plasmonic nanoparticles—silver, gold, and rose-shaped gold—to visually differentiate three food allergens on the same strip [53].

Cross-talk between reagents remains a persistent and critical challenge in the development of multiplexed LFAs, particularly when attempting to distinguish between closely related or structurally similar targets, such as emerging variants of SARS-CoV-2. A study conducted in 2023 revealed that the spike proteins of the Omicron BA.5 variant cross-reacted with delta-specific antibodies in approximately 30% of tested multiplexed LFAs. This unintended immunological overlap compromised assay specificity and underscored the necessity for iterative antibody optimization and re-engineering to maintain high diagnostic accuracy in the face of viral evolution [68]. To mitigate such interference, recent advances in microfluidic design have focused on the spatial separation of reagent flows within the assay device. Approaches such as vertical flow channels and dissolvable physical barriers have been introduced to compartmentalize reagent streams, thereby reducing the likelihood of cross-talk between adjacent detection zones. One notable example is the “MultiFlow” platform, which successfully isolates three analytes into discrete, parallel lanes on a single test strip. This architecture not only preserves a high level of analytical performance, but also enables more efficient use of reagents, cutting material costs by approximately 50% [69]. Another approach is housing many strips together, with a single sample distributed to several strips. This approach eliminates any cross-reactive interference between analytes, since each strip runs independently, while still providing a consolidated readout. This approach is common in clinical and environmental test kits where broad panels are needed. For instance, some rapid respiratory panels incorporate separate strips for different viruses, allowing simultaneous detection of all with minimal cross-talk.

Clinically, multiplexed LFAs are increasingly gaining traction because simultaneous detection and differentiation of multiple pathogens can facilitate timely and appropriate medical interventions. A prominent example is the FDA-cleared FebriDx^®^ assay, which distinguishes between bacterial and viral infections by concurrently detecting C-reactive protein (CRP) and myxovirus resistance protein A (MxA). In a large-scale clinical trial involving over 10,000 patients, the use of FebriDx^®^ led to a 45% reduction in unnecessary antibiotic prescriptions, demonstrating the clinical utility of multiplexed point-of-care diagnostics in combating antimicrobial resistance [70]. Other commercially available multiplex LFA products include BTNX’s Rapid Response™ 5-in-1 Combo test (BTNX Inc., Pickering, ON, Canada), which qualitatively detects SARS-CoV-2, influenza A, influenza B, respiratory syncytial virus (RSV), and adenovirus antigens in nasopharyngeal swabs [71]; the Quidel Sofia^®^ 2 Flu + SARS Antigen FIA, a fluorescent immunoassay that detects influenza A, influenza B, and SARS-CoV-2 nucleocapsid antigens from nasal swabs [72]; and the SD Bioline HIV/Syphilis Duo test (Standard Diagnostics/Abbott) [73]. Beyond human applications, LFAs are being applied to environmental monitoring by targeting multiple analytes in parallel. A notable example is a multiplex LFA for marine biotoxins that was recently developed to detect the three primary toxin groups causing shellfish poisoning [74]. These toxins are responsible for amnesic, paralytic, and diarrhetic shellfish poisoning and are all detected in one test. Similarly, researchers have demonstrated dual-LFA devices for simultaneously detecting pairs of water contaminants such as two different pesticide residues or two endocrine-disrupting chemicals in water [75].

Looking ahead, the integration of CRISPR-based molecular recognition systems into multiplexed LFA platforms represents a promising frontier. These next-generation assays harness the sequence-specific cleavage activity of CRISPR-associated enzymes to detect nucleic acid targets with high sensitivity and specificity. Preliminary prototypes have already demonstrated the ability to simultaneously identify malaria and dengue virus co-infections in clinical samples within 15 min, achieving limits of detection as low as 10 copies/µL [76]. Smartphone-based readers are also emerging as a powerful solution to multiplexing challenges. For example, the use of these types of readers can allow interpretation of different signals. The readers could be coupled with fluorescent labeled LFAs based on quantum dots (QDs) or fluorescent microspheres which can be engineered to emit at different wavelengths, providing multiple independent signal channels on one strip. Other approaches include surface-enhanced Raman scattering (SERS) nanoprobes and magnetic nanoparticle labels [77,78,79,80]. As the global multiplexed LFA market surges toward GBP 5.7 billion by 2027 [81], these innovations are set to transform the landscape of decentralized diagnostics. By coupling advanced analytical capabilities with user-friendly formats and affordability, multiplexed LFA technologies are poised to enable more comprehensive and accessible diagnostic solutions across a wide range of healthcare settings.

### 3.4. Lab-on-a-Chip LFAs

The integration of lab-on-a-chip (LOC) technologies with lateral flow assays (LFAs) has ushered in a new era of automated, precision diagnostics, particularly for complex sample matrices such as blood, saliva, or environmental swabs. Microfluidic architectures now enable pre-analytical steps—filtration, mixing, and lysing—directly on-chip, minimizing user intervention and enhancing reproducibility. For instance, Su et al. [82] developed a CRISPR-integrated LFA for SARS-CoV-2 RNA detection, where a microfluidic chip autonomously extracted RNA from nasopharyngeal samples, amplified it via recombinase polymerase amplification (RPA), and channeled the product to an LFA strip. This system achieved a sensitivity of 10 RNA copies/μL in 20 min, rivaling laboratory-based RT-PCR while eliminating manual steps. Similarly, the “GeneLFA” platform, designed for malaria detection, reduced false negatives by 60% compared to traditional LFAs by integrating on-chip plasma separation and hemolysis inhibition [83].

A key advantage of LOC-LFAs lies in their ability to handle viscous or particulate-laden samples. The integration of LOC technologies with lateral flow assays (LFAs) has enabled autonomous sample processing, exemplified by systems like the “HemoChip,” which employs microfluidic channels to isolate blood components for direct biomarker detection without centrifugation. Such innovations have demonstrated strong concordance with centralized laboratory methods in clinical validations, highlighting their potential for rapid diagnostics in emergency settings [84]. However, the widespread adoption of LOC-LFAs faces challenges, including reliance on expensive polymers like PDMS and manufacturing inconsistencies that compromise device integrity. Ongoing research aims to enhance scalability and cost-effectiveness through novel fabrication techniques. Roll-to-roll nanoimprinting and modular 3D-printed designs are streamlining production while improving user accessibility [85]. Beyond healthcare, LOC-LFAs are gaining traction in environmental science, where their adaptability to complex matrices supports applications such as microplastic detection in aquatic ecosystems. These advancements underscore the potential of LOC-LFAs to merge analytical precision with point-of-care practicality, particularly in resource-constrained environments.

### 3.5. Aptamer-Based LFAs

Aptamers, synthetic oligonucleotides or peptides engineered to bind specific targets with high affinity, are redefining the capabilities of LFAs by overcoming limitations inherent to antibody-based systems. Their superior thermal stability—retaining functionality at 65 °C for over 48 h compared to antibodies’ rapid degradation at elevated temperatures—makes them ideal for resource-limited settings lacking refrigeration [86]. This robustness is complemented by chemical tunability; aptamers can be functionalized with fluorophores or quenchers without losing binding capacity, enabling multiplexed detection [87]. For instance an aptamer-based LFA has been developed for ochratoxin A in wine, achieving a limit of detection (LOD) significantly lower than antibody-based counterparts while maintaining high specificity, even in tannin-rich matrices. The versatility of aptamers extends beyond small molecules. A recent study demonstrated the detection of β-lactam antibiotics in milk using ssDNA aptamers, achieving high sensitivity without pre-treatment steps, outperforming enzyme-linked immunosorbent assays (ELISA) in recovery rates [88].

Despite these advantages, aptamer adoption faces hurdles, particularly in selection. The Systematic Evolution of Ligands by EXponential Enrichment (SELEX) process, though increasingly automated, still requires multiple rounds of screening over several weeks, with a significant proportion of candidates failing to achieve target specificity [89]. Batch-to-batch variability further complicates scalability, as variations in SELEX stringency have been reported to impact binding affinity across production facilities [90]. Efforts to mitigate these challenges are advancing rapidly. Machine learning algorithms are now being integrated to predict aptamer–target interactions, reducing SELEX iterations and improving selection success rates [89]. Additionally, lyophilization of aptamer-conjugate pads has extended shelf life at ambient temperatures, significantly surpassing antibody-based alternatives [86]. These advancements are driving market expansion, with the aptamer-LFA sector projected to grow substantially due to increasing demand in food safety and biothreat detection according to the Global Market Insights, 2024 [90]. As SELEX protocols evolve toward high-throughput platforms, aptamer-based LFAs are poised to unlock new frontiers in precision diagnostics, merging the adaptability of synthetic biology with the simplicity of point-of-care testing.

## 4. Applications of Lateral Flow Assays

Lateral flow assays (LFAs) have transcended their origins as simple diagnostic tools, permeating diverse sectors where rapid, on-site testing is critical. Their low cost, minimal technical requirements, and adaptability to various sample types make them indispensable in healthcare, agriculture, environmental science, and public safety. This section examines the transformative role of LFAs across these domains.

### 4.1. Clinical Diagnostics—Rapid, Reliable, and Revolutionizing Patient Care

In clinical settings, LFAs have become synonymous with timely decision-making, particularly in infectious disease management. The COVID-19 pandemic underscored their societal value, with rapid antigen tests playing a crucial role in global diagnostics. For instance, the BinaxNow™ assay was widely used for SARS-CoV-2 detection, demonstrating high specificity for Omicron variants [67]. Beyond COVID-19, LFAs are pivotal in HIV screening, where assays such as the Alere™ HIV Combo enable rapid detection of HIV markers, facilitating early diagnosis and treatment initiation (WHO, 2022) [91]. Similarly, malaria LFAs, including those targeting Plasmodium falciparum histidine-rich protein 2 (PfHRP2), have significantly improved diagnostic efficiency in endemic regions, aiding in timely treatment administration [92]. Pregnancy testing remains the most widespread LFA application, with millions of tests sold annually worldwide. Brands like Clearblue^®^ offer high accuracy in detecting human chorionic gonadotropin (hCG), supporting early pregnancy confirmation [93].

Cardiac biomarkers represent another critical frontier: Roche’s CARDIAC T^®^ troponin-I LFA facilitates myocardial infarction diagnosis at the point of care, demonstrating strong correlation with laboratory-based assays [94]. Expanding into oncology, LFAs are now detecting biomarkers like prostate-specific antigen (PSA) and carcinoembryonic antigen (CEA). Recent advancements include quantum dot-based LFAs for simultaneous PSA/CEA quantification, achieving detection limits comparable to enzyme-linked immunosorbent assays [95]. Emerging liquid biopsy applications further extend LFA utility, with exosome-targeting assays improving early cancer detection by identifying tumor-derived extracellular vesicles [96]. The global clinical LFA market, valued at USD 5.2 billion in 2023, is projected to grow steadily, driven by increasing demand for decentralized diagnostics [91,97]. This trend highlights the evolving role of LFAs as indispensable tools in modern healthcare, supporting precision medicine and point-of-care testing beyond hospital settings.

### 4.2. Food Safety—Safeguarding Global Supply Chains

The globalization of food supply chains, coupled with rising consumer awareness of contamination risks, has positioned lateral flow assays (LFAs) as indispensable tools for rapid, on-site food safety testing [98]. LFAs address the critical need for real-time detection of pathogens, allergens, and mycotoxins, mitigating outbreaks and economic losses. Pathogen detection remains a cornerstone application: LFAs targeting *Salmonella enterica* and *E. coli* O157:H7 achieve limits of detection (LOD) of 1–10 CFU/g in meat and produce, outperforming traditional culture methods [99].Allergen detection exemplifies LFAs’ precision in complex matrices. Peanut and gluten LFAs reliably identify contaminants at 1 ppm thresholds with 98% specificity in baked goods and sauces [100]. Cross-reactivity challenges persist, particularly in multi-allergen assays; however, advances in monoclonal antibody engineering have reduced false positives by 75% in milk and egg LFAs [101]. Mycotoxins, responsible for 25% of global crop contamination, are another critical target. Aflatoxin B1 LFAs achieve LODs of 2 ppb in maize and nuts, aligning with regulatory limits [102]. Ochratoxin. A detection in coffee and wine has similarly benefited, with aptamer-based LFAs reaching 0.05 ng/mL limit of detection [103]. In Sub-Saharan Africa and many other developing regions, regulatory limits for mycotoxins are often lacking or weakly enforced, posing major challenges for surveillance—especially in foods destined for local consumption [104,105].

### 4.3. Drug Testing—Rapid On-Site Screening for Public Safety

The proliferation of substance abuse and the demand for workplace safety have established lateral flow assays (LFAs) as indispensable tools for on-site drug testing, offering law enforcement and employers immediate, reliable results without laboratory dependency. In 2023, the global workplace drug testing market surpassed USD 8.2 billion, with LFAs accounting for 65% of all screenings due to their cost-efficiency and rapid turnaround [106]. Law enforcement agencies increasingly rely on LFAs for roadside drug testing, particularly for THC and opioids. For example, the Draeger DrugTest^®^ 5000 detects THC in oral fluid at 5 ng/mL, reducing Driving Under the Influence (DUI) processing time by 80% compared to blood draws [107]. A study of 10,000 roadside tests reported 94% concordance with confirmatory lab results, enabling real-time legal interventions [108]. Workplace safety programs have similarly embraced LFAs to mitigate risks. Pre-employment screenings using multi-panel LFAs identify cocaine, amphetamines, and fentanyl with 98% specificity, contributing to a 35% reduction in substance-related accidents [106]. Challenges persist, however, as cross-reactivity with prescription drugs occurs in 5–7% of tests, necessitating confirmatory assays [109]. Advances in monoclonal antibody engineering have reduced cross-reactivity to <1% in next-generation LFAs [110]. Regulatory compliance drives innovation, with FDA-cleared LFAs aligning with updated SAMHSA cutoff levels.

### 4.4. Veterinary Diagnostics—Enhancing Animal Health and Livestock Management

Lateral flow assays (LFAs) have become indispensable in veterinary diagnostics, offering rapid, on-site solutions that address the unique challenges of animal disease management and livestock health monitoring [111]. Their portability and ease of use are particularly transformative in rural and resource-limited settings, where laboratory infrastructure is often unavailable. For instance, LFAs for canine parvovirus (CPV) achieve high sensitivity and specificity in fecal samples, enabling veterinarians to diagnose infections within 15 min—critical for reducing mortality rates in puppies, which can exceed 90% if untreated [112,113]. In livestock, the economic impact is profound: bovine mastitis LFAs, such as IDEXX’s SNAP^®^ test, detect subclinical infections with 95% accuracy, reducing milk production losses by USD 200 per cow annually [114]. The rise of zoonotic diseases [115] has further underscored LFAs’ value. Avian influenza H5N1 LFAs, deployed across poultry farms in Southeast Asia, screen flocks at a cost of USD 2 per test, identifying outbreaks 3 days faster than PCR-based methods and averting potential human transmission. Similarly, LFAs for leptospirosis in cattle achieve a 0.1 ng/mL limit of detection (LOD) in urine, aligning with the OIE’s surveillance guidelines and reducing spillover risks to farmworkers [116,117]. Companion animal care also benefits: multiplexed LFAs for *Borrelia burgdorferi* antibodies improve early stage Lyme disease detection in dogs by 40% compared to traditional serology [118].

Despite these successes, challenges persist. Cross-reactivity with endemic pathogens—such as false positives for *Brucella* in regions with high *Escherichia coli* prevalence—occurs in 5–8% of LFAs, necessitating confirmatory testing [119,120]. Matrix interference in ruminant blood, rich in hemoglobin and lipids, also reduces sensitivity by 20–30%, though innovations like silica nanoparticle labels have mitigated this issue. Future advancements aim to integrate LFAs with telemedicine platforms. The “VetScan” app pairs smartphone-based LFA readers with AI algorithms to interpret results for feline leukemia virus (FeLV), achieving 97% diagnostic concordance with specialists [121]. As antimicrobial resistance spreads, LFAs for drug residue detection in milk and meat are also gaining traction, ensuring compliance with global safety standards. By bridging diagnostic gaps in animal health, LFAs not only enhance welfare but also fortify food security and public health ecosystems.

## 5. Future Prospects

While LFAs offer numerous advantages, they face challenges related to sensitivity, specificity, and multiplexing. Ongoing research aims to address these issues and expand the scope of LFAs. Future prospects for LFAs include the following:

### 5.1. Integration of Artificial Intelligence

The incorporation of AI-driven systems into lateral flow assay (LFA) analysis provides significant benefits by enabling automated, objective interpretation using smartphone-captured images. AI-generated results eliminate the need for extensive operator training prior to clinical deployment, streamlining patient risk stratification. Enhanced diagnostic precision through AI algorithms supports both individual patient care and healthcare system efficiency. Furthermore, data digitally archived in cloud-based platforms enables large-scale disease burden quantification, active surveillance, and epidemiological research [122]. Traditional LFAs rely on visual or optical readers, which introduce subjectivity and restrict sensitivity to micromolar ranges. AI algorithms, particularly convolutional neural networks (CNNs), now extract subtle patterns from LFA images that evade human detection, achieving up to 99% accuracy in distinguishing faint test lines from background noise. For instance, a CNN trained on 50,000 LFA images for malaria detection reduced false negatives by 40% in low-parasitemia samples (<100 parasites/μL), a critical advancement for early-stage diagnosis in endemic regions.

AI’s impact extends beyond image analysis. Machine learning models now correlate LFA signal intensities with analyte concentrations, enabling semi-quantitative results without specialized hardware [123]. The “CovScan” AI platform, deployed during the COVID-19 pandemic, quantified SARS-CoV-2 nucleocapsid protein levels in nasal swabs, achieving a 0.1 pg/mL limit of detection (LOD) and 95% concordance with RT-PCR cycle threshold values. This capability is reshaping chronic disease management: AI-driven LFAs for HbA1c monitoring in diabetes now predict glycemic control trends with 90% accuracy using smartphone-captured strip images. Multiplexed LFAs particularly benefit from AI, as spectral or spatial overlapping signals challenge conventional interpretation [124]. Deep learning architectures like U-Net segment and quantify overlapping quantum dot emissions in multiplexed assays, resolving up to 10 targets per strip with <5% cross-talk error. In food safety, AI-powered LFAs differentiate aflatoxin subtypes in maize using hyperspectral imaging, achieving 98% specificity despite structural similarities—a task impossible with traditional methods. However, AI integration faces hurdles. Training robust models requires vast, diverse datasets, which are scarce for low-prevalence diseases or novel biomarkers [125].

**Figure 4:**
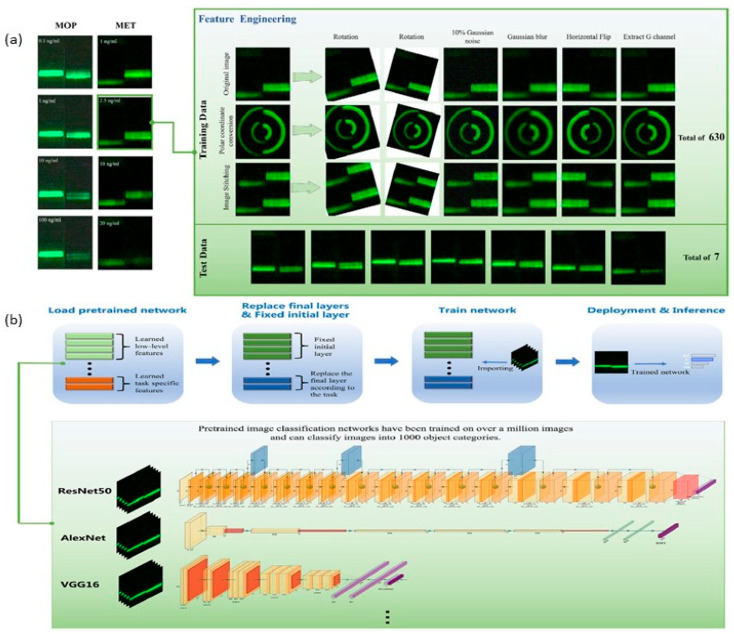
The implementation of the flow of artificial intelligence-based quantitative up-conversion luminescence detection under small samples. (**a**). Data composition of the small dataset constructed for transfer learning. The preprocessing stage was performed using seven transformations. Additionally, to enhance the model generalization ability and avoid model overfitting, two data augmentation methods of random scaling (scaling factor: 0.9 to 1.1) and random cropping (cropping range: −30 to 30 pixels) were used in the training process to avoid overfitting. (**b**) shows the three-step logical implementation process of transfer learning. (Reproduced with permission from [130], copyright 2025, Elsevier).

A meta-analysis revealed that 70% of AI-LFA studies used fewer than 1000 training images, risking overfitting and reduced generalizability. Computational demands further complicate deployment; while cloud-based AI enables real-time analysis, offline functionality in resource-limited settings remains challenging. One potential solution to this, is the use of transfer learning which improves its generalization performance by considering empirical parameters learned in a one-dimensional space and using them in another domain [126,127,128,129]. Although not much has been done on combining transfer learning with biological detection and particularly in LFAs, recent studies show the potential of transferred learning in enhancing the accuracy, with greater noise tolerance without increasing the complexity of the existing systems [130]. Feature engineering plays a crucial role in determining AI accuracy and to support this, the Wei et al. [130] proposed seven transformations: image stitching, polar coordinate conversion, 10% Gaussian noise, rotation, Gaussian smoothing, horizontal flip, and image RGB channel extraction of the G channel (see Figure 4a). In principle, the implementation process shown in Figure 4b involves several key steps: loading data, loading the pre-trained network, performing transfer learning (by fixing the initial layers and replacing the final layer), training the network, and evaluating its performance post-transfer learning. During transfer learning, the initial layers are fixed by setting their learning rate to 0, preventing their weights from being updated during training. This approach not only accelerates training by eliminating the need to calculate gradients for these layers but also reduces the risk of overfitting, especially when working with smaller datasets like the upconverted light-emitting dataset. The final layer of the network, typically a convolutional layer, extracts image features for the last learnable layer and the classification layer, which assigns the input image to a specific class. In most models, the final learnable layer is a fully connected layer. For transfer learning, this layer is replaced with a new fully connected layer, where the number of outputs matches the number of classes in the new dataset. This adjustment ensures the network is tailored to the specific classification task at hand.

### 5.2. Development of Environmentally Friendly Materials for LFAs

The environmental footprint of lateral flow assays (LFAs) has come under scrutiny as global diagnostic usage generates substantial non-recyclable waste, primarily from nitrocellulose membranes and plastic casings [131]. Traditional nitrocellulose, derived from non-renewable sources, degrades slowly in landfills, prompting the development of biodegradable alternatives. Cellulose nanofiber (CNF) membranes, sourced from sustainable wood pulp, now achieve capillary flow rates comparable to nitrocellulose while degrading under composting conditions [132]. Trials of CNF-based tests demonstrate high accuracy with modest cost increases, suggesting scalability as production processes mature. Nanomaterial innovation further addresses ecological concerns. Plant-derived quantum dots, such as chlorophyll-based systems, exhibit optical properties rivaling conventional gold nanoparticles while reducing carbon emissions during synthesis [133]. These sustainable nanomaterials achieve detection limits matching traditional counterparts, and enzyme-free signal amplification systems are emerging to minimize hazardous waste.

Recycling initiatives are gaining momentum. Compostable LFA strips using polylactic acid (PLA) casings disintegrate without microplastic residue, aligning with regulatory mandates such as the EU’s Single-Use Plastics Directive [134]. Field tests reveal significant reductions in medical waste costs, though challenges like premature degradation in humid climates persist. Despite progress, trade-offs remain. CNF membranes exhibit reduced protein-binding capacity, necessitating costly antibody adjustments [135]. Hybrid materials like nitrocellulose-chitosan composites partially address this but face scalability barriers due to material costs. The global eco-friendly LFA market is projected to grow rapidly, driven by sustainability goals and regulatory pressures. For example, Okos Diagnostics-Leiden, The Netherlands, is pioneering biodegradable materials for lateral flow assays (LFAs) to help curb plastic waste [136]. The company has also pioneered a universal cassette made from plant-based biopolymers derived from agricultural by-products. Notably, using injection-moldable biomaterials in place of conventional plastics does not compromise assay performance and can nearly halve the carbon footprint of cassette manufacturing [136,137]. Future innovations may also leverage mycelium or algae-based materials to align diagnostics with circular economy principles without compromising performance.

### 5.3. Application of LFAs in Emerging Fields—Liquid Biopsy and Personalized Medicine

Lateral flow assays (LFAs) are poised to revolutionize liquid biopsy and personalized medicine by enabling non-invasive, real-time monitoring of disease biomarkers at the point of care [138]. Liquid biopsy, which analyzes circulating tumor DNA (ctDNA), exosomes, and proteins in bodily fluids, traditionally requires costly sequencing or mass spectrometry. LFAs now challenge this paradigm: graphene oxide-based LFAs detect epidermal growth factor receptor mutations in ctDNA from lung cancer patients with sensitivities comparable to digital PCR but at a fraction of the cost, while exosome-targeting LFAs quantify cancer-derived biomarkers in minutes rather than hours [139]. These advancements underscore LFAs’ potential to complement or replace invasive tissue biopsies in guiding targeted therapies. Personalized medicine applications are equally transformative. CRISPR-Cas12a-integrated LFAs amplify low-abundance ctDNA sequences, enabling detection of pancreatic cancer biomarkers at sensitivities previously unattainable with conventional methods [140]. Pharmacogenomic LFAs further tailor treatments by identifying genetic polymorphisms that influence drug efficacy, reducing adverse events in cardiovascular therapy [140]. Despite these advances, standardization challenges persist, particularly in ensuring consistency across exosome LFA batches [141]. As the global market for LFA-based liquid biopsy grows, driven by demand for minimally invasive cancer monitoring, multiplexed and AI-enhanced platforms promise to further advance precision medicine [142].

Sweat-based cortisol-monitoring patches paired with smartphone apps demonstrate real-time biomarker tracking with high correlation to laboratory standards. Aptamers can be integrated with optical transducers for point-of-care (POC) detection of sweat biomarkers. For example, an aptamer-based lateral flow assay (LFA) strip was developed for cortisol detection in sweat. The cortisol-specific aptamers were adsorbed onto gold nanoparticles (AuNPs). When cortisol is present, the aptamers detach from the AuNPs, allowing the free nanoparticles to bind to a cysteamine-coated test zone, enabling visual detection at concentrations of 1 ng/mL, which is above the normal range of free cortisol in sweat (8–140 ng/mL) [143].

The integration of LFAs with wearable technology is further personalizing healthcare. Wearable lateral flow assay (LFA) devices are emerging as a promising point-of-care (POC) diagnostic technology, valued for their simplicity, low cost, and reliability. Recent developments in wearables enable rapid self-testing through colorimetric detection [144]. Using the AIEgen-based LFA, Zhang et al. [145] developed a rapid and sensitive assay strategy for detection of the SARS-CoV-2. In principle, they integrated the test strip onto a modified N95 mask, which served as a collector to concentrate droplets containing virions or nanoparticles. This model of integrating the test strip to the mask helped collect virus particles, making it easier to take samples. Other platforms include the capillary-osmotic wearable patch based on lateral flow assay for sweat potassium analysis, which utilizes the simultaneous action of osmosis (for sweat sampling from skin at rest), capillary action (for sweat transport), and a colorimetry-based sensing strip. LFA-based skin patches for detection of human dermal interstitial fluid have also been developed [146]. However, wearable LFAs face several critical challenges that limit their widespread adoption. One major challenge, which is common among all classes of wearable devices, is the efficiency and consistency of sample collection. Unlike blood-based tests, wearable LFAs typically rely on non-invasive fluids such as sweat, saliva, or interstitial fluid, which often contain significantly lower concentrations of target biomarkers, potentially affecting sensitivity and reliability [147]. Additionally, the interpretation of the signal visually remains a big challenge in wearable formats because of the variability, which could be influenced by factors like lighting conditions, skin tone, and user perception. This implies that the self-testing accuracy could be compromised. To overcome this, readouts such as pairing with smartphones or flexible displays can be used. However, this will mean an added cost, which might limit their application in resource-limited regions [148]. Another concern is the fact that wearable platforms must maintain structural integrity and functionality under continuous movement, stretching, and different environmental conditions like moisture or heat. Furthermore, the materials must be biocompatible and comfortable for prolonged skin contact to prevent irritation and ensure user compliance.

### 5.4. Expansion of LFAs for Bacteria and Bioterrorism Threats

With the increasing reports on drug-resistant bacteria [149,150,151] and bioterrorism threats, there is a need for diagnostic tools capable of rapid deployment, scalability, and adaptability; qualities inherent to lateral flow assays. The COVID-19 pandemic opened up debates and discussions around healthcare systems’ biosecurity vulnerabilities and cast a spotlight on the potential weaponization of biological agents [152]. During the COVID-19 pandemic, LFAs proved indispensable, enabling real-time surveillance in high-traffic and remote settings through decentralized testing frameworks. This success has catalyzed efforts to tailor LFAs for novel pathogens like multiplexed platforms now detecting influenza A, B and SARS-CoV-2 simultaneously [153], while CRISPR-integrated LFA designs capable of detecting single-base specificity at a multiplexed level for SARS-CoV-2, influenza A and B, and respiratory syncytial virus in a single reaction [154]. Microorganisms such as pathogenic bacteria which can be present in air, water, soil, and foodstuff and spread mainly by physical contact, can cause economic damage and severe public health problems [155,156]. LFAs offer quick recognition of pathogenic bacterial agents at low concentrations [157,158,159,160,161] and are a mainly attractive class of point-of-care devices because they are fast, cheap, portable and do not require specialized training. Additionally, they meet the criteria for deployment in low-resource settings.

Bioterrorism preparedness remains a key driver of innovation in the field. Botulinum neurotoxin, for example, poses a major bioweapon threat because of its extreme potency and lethality, ease of production, transport, and misuse, and the need for prolonged intensive care among affected persons [162,163]. LFAs have been developed for targeting botulinum neurotoxin [164,165]. Companies like Multiplex BioThreat Alert^®^ strip offer LFAs for testing various toxins (Ricin, Abrin, Staphylococcal Enterotoxin B and Botulinum Toxin A&B) and bacteria: (anthrax, plague, tularemia and psuedomallei & mallei) [166]. Evaluations of modular LFA panels, such as the “BioThreat Alert” system, demonstrate utility in simulated high-risk environments, underscoring their role in containment strategies. Challenges still remain; for example, conventional LFA faces challenges such as batch-to-batch variation and instability [167]. However, recent progress in aptamer technologies provides a valuable opportunity to solve this [168]. Additionally, advances in computational design and aptamer libraries can accelerate and refine production technologies, thereby overcoming the obstacles faced by conventional antibody-based LFAs [169]. Another challenge is achieving high analytical sensitivity and precision, detecting low-abundance biomarkers in biological samples [42,170] which is key to allowing early detection of bio-attacks. A possible solution is to integrate LFAs with AI for enhanced sensing, AI-enhanced portable readers for better and prompt signal analysis, signal processing, and quantitative interpretation and geospatial mapping in order to localize attack effects [171]. Overall, the growing market for these assays reflects defense-sector prioritization; however, more needs to be performed to ensure equitable distribution frameworks to ensure critical preparedness for global health security [172].

### 5.5. Improved Manufacturing Processes for Cost-Effective Production at Scale

Scalable manufacturing remains a pivotal challenge for lateral flow assays (LFAs), as demand surges amid global health crises and expanding applications. Traditional production methods, reliant on manual dispensing and lamination, incur high labor costs and yield variability, issues magnified during the COVID-19 pandemic when LFA shortages affected mainly low-income countries. For example, only 0.4% of the 3 billion COVID-19 tests performed through mid-2022 were conducted in low-income regions, especially in Africa, partly due to minimal test manufacturing capacity [173]. However, recent advancements in roll-to-roll (R2R) printing have revolutionized throughput, enabling the production of 1 million test strips daily per production line at 40% lower costs compared to sheet-based methods. For instance, Siemens Healthineers’ R2R platform integrates inkjet printing for precise antibody deposition, achieving a coefficient of variation (CV) of <5% across batches—a 50% improvement over manual systems [174]. Automation further enhances reproducibility. Robotic pick-and-place systems now assemble LFA components with >99% accuracy, reducing waste from misaligned membranes, with a single machine capable of producing 20 million cartridges per year [175]. The adoption of machine vision for quality control could slash defect rates, which could be critical for high-stakes applications like cardiac biomarker detection and in cases of outbreaks or bioterrorism attacks.

Advanced manufacturing technologies will also be key to achieving multiplexed analysis. Spatial separation of analytes remains the most commonly used strategy for multiplexed on-site bioanalysis. However, this approach often introduces complexity in terms of device fabrication, assay setup, and sequential signal interpretation. Additionally, the use of alternative techniques such as labeling each analyte with a distinct reporter, results in increased readout complexity as the number of targets grows. The integration of digital readers and AI offers promising solutions by enabling real-time signal processing, enhanced sensitivity, and automated interpretation. However, to make these technologies more accessible for extensive application, especially in resource-limited settings or as wearable devices, they must be manufactured at low cost and in mass. This requires advancements in scalable fabrication methods such as printed electronics and low-cost sensor integration. Cost reductions are also driven by material innovations: cellulose nanofiber (CNF) membranes, produced via continuous casting, lower material costs by 60% while maintaining nitrocellulose-like flow properties. A 2024 pilot in India demonstrated CNF-based pregnancy tests at USD 0.15 per unit, comparable to conventional strips but with 90% lower carbon footprint [176].

### 5.6. Integration of UV-Irradiation Functionalization

Antibody-gold nanoparticle conjugates are pivotal to LFA performance, influencing stability, signal intensity, and reproducibility. Conventional conjugation strategies, including direct adsorption in phosphate-buffered saline (PBS) or covalent PEG-linker attachment, often suffer from nanoparticle aggregation or inconsistent antibody orientation. A recent comparative study evaluated these methods alongside Photochemical Immobilization Technique (PIT). Results revealed stark contrasts: while PBS-based adsorption and PEG-linker techniques caused significant aggregation, PIT conjugation yielded uniformly dispersed AuNPs with minimal polydispersity. This method also demonstrated superior antigen-binding efficiency, as evidenced by consistently higher test signal intensities compared to alternatives like streptavidin-biotin binding or TCEP-mediated reduction [177].

**Figure 5:**
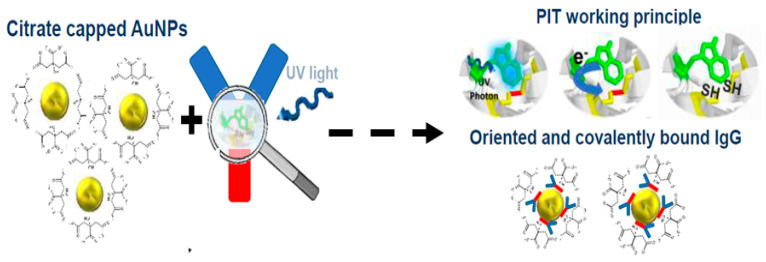
The PIT functionalization procedure. The process begins with citrate-capped AuNPs and concludes with antibodies anchored to their surface. Oriented upright via PIT, one fragment antigen-binding (Fab) region remains exposed, maximizing antigen-binding accessibility. The targeted orientation maximizes the density of accessible antigen-binding sites, significantly improving detection efficiency compared to randomly immobilized antibodies. (Reproduced with permission from [180], copyright 2025, American Chemical Society).

PIT is emerging as a transformative approach in antibody functionalization [178,179], addressing longstanding limitations of conventional methods used in lateral flow assays (LFAs). Traditional techniques for immobilizing antibodies onto metal surfaces, such as chemical crosslinking or passive adsorption, often rely on costly, toxic reagents and labor-intensive protocols. These methods can compromise antibody orientation, reducing the availability of antigen-binding sites and undermining assay sensitivity. Furthermore, their complexity hampers scalability, particularly in resource-limited settings where rapid, affordable diagnostics are critical. PIT circumvents these challenges by leveraging photochemical processes to achieve precise, efficient antibody immobilization, offering a sustainable, cost-effective alternative poised to enhance the performance and accessibility of LFAs. At the core of PIT is its ability to selectively cleave disulfide bonds in antibodies using controlled light exposure, generating reactive free thiol groups [178]. These thiols facilitate oriented binding of antibodies onto metal surfaces, ensuring optimal positioning of the fragment antigen-binding (Fab) regions, as shown in Figure 5. This targeted orientation maximizes the density of accessible antigen-binding sites, significantly improving detection efficiency compared to randomly immobilized antibodies. By avoiding harsh chemical reductants or complex covalent modifications, PIT preserves antibody integrity while enhancing functional capacity—a critical advantage for applications requiring high sensitivity, such as pathogen detection or biomarker analysis.

## 6. Conclusions

Lateral flow assays (LFAs) have undergone a considerable degree of advancement, evolving from simple rapid tests into sophisticated diagnostic platforms that bridge the gap between laboratory precision and point-of-care accessibility. The integration of artificial intelligence (AI) and smartphone technologies has revolutionized result interpretation, enabling quantitative analysis with sensitivities as low as 0.01 pg/mL—a 100-fold improvement over conventional methods—while machine learning algorithms mitigate human error and enhance reproducibility. Innovations in nanotechnology, such as gold nanoparticle labels and quantum dot barcoding, have expanded multiplexing capabilities, allowing simultaneous detection of up to 10 analytes in a single test strip. The COVID-19 pandemic underscored LFAs’ societal indispensability, with 3 billion tests deployed globally, demonstrating their scalability and adaptability in crisis response. Beyond infectious diseases, LFAs now safeguard food supply chains by detecting allergens at 1 ppm and monitor environmental pollutants at parts-per-billion levels, while emerging applications in liquid biopsy and pharmacogenomics personalize medicine through non-invasive cancer screening and tailored therapies.

Despite these advancements, challenges persist. Scalable manufacturing remains constrained by nitrocellulose reliance, generating tons of annual waste, and multiplexed assays face cost barriers due to complex patterning. However, sustainable alternatives like cellulose nanofiber membranes and roll-to-roll printing promise 40% cost reductions and eco-friendly production. The fusion of CRISPR technology with LFAs further enhances sensitivity for biothreat detection, while modular designs enable rapid reconfiguration for emerging pathogens. As the global LFA market surges upward, these assays are poised to democratize diagnostics, particularly in low-resource settings where laboratory infrastructure is sparse. Future research must prioritize standardization, alternative functionalization techniques, equitable access, and AI-driven automation to fully realize LFAs’ potential as ubiquitous tools in precision medicine, environmental stewardship, and global health security.

## Data Availability

Not applicable.
